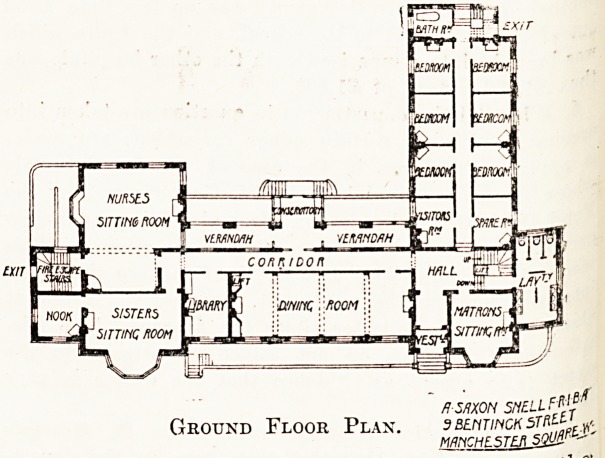# Fulham Parish Infirmary Nurses' Home

**Published:** 1913-05-03

**Authors:** 


### HOSPITAL ARCHITECTURE AND CONSTRUCTION.

### Fulham Parish Infirmary Nurses' Home.

In no way has the development of the work of Poor-Law infirmaries shown more marked improvement than in the provision of suitable quarters for the nursing staff. The latest example in London is the Nurses' Home for the Fulham Parish Infirmary, which we illustrate to-day. The new building is on the north side of the Fulham Road, immediately opposite the infirmary, with which it is connected by a subway under the road. It contains accommodation for the matron, seven sisters, and fifty-two nurses.

First Floor Plan.

In the basement are the kitchen offices, stores, linen room, bicycle house, etc., the bicycle house being approached by a sloping way from the outside.

On the ground floor are common sitting rooms for sisters and for nurses, a small library, the dining room, matron's sitting room, seven bedrooms, and a visitors' room. At the back a verandah is arranged facing the garden, and a small conservatory through which the garden is approached. The verandah, which faces north, will provide a cool retreat in summer. There are three staircases, the principal one being at the east end, with a lift in the well-hole. At the west end of the front block is an escape staircase, while a secondary staircase also available as exit in case of fire is at the north end of the east wing.

Ground Floor Plan.

The two upper floors are occupied by bedrooms, each one being provided with a fireplace. There is a fair supply of bath rooms, but for so large a number of inmates the provision of w.c.s and lavatories seems inadequate.

The building was designed by Mr. A. Saxon Snell, F.R.I.B.A., and the cost was rather over £12,000.

## Figures and Tables

**Figure f1:**
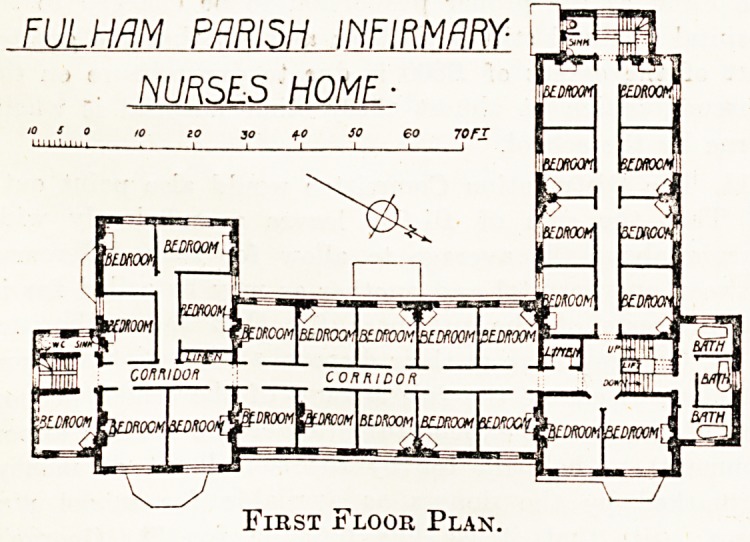


**Figure f2:**